# Gallbladder schistosomiasis: rare but possible, a case report and review of the literature

**DOI:** 10.11604/pamj.2019.32.91.17907

**Published:** 2019-02-26

**Authors:** Mohamed Hedfi, Mehdi Debaibi, Senda Ben Iahouel, Adnen Chouchen

**Affiliations:** 1 Surgery Department, Fsi Hospital Marsa, Tunisia

**Keywords:** Gallbladder, schistosomiasis, treatment

## Abstract

After Malaria, schistosomiasis remains the most important tropical disease in large parts of the world. It affects mainly the colon and the urinary tract. The hepatic involvement is significantly frequent, particularly by the mansoni species. Still one of the extremely rare locations is the gallbladder. Our case is about a 51 year old woman from Tunisia, which is no longer considered an endemic country, with no particular medical history, underwent surgery for symptomatic cholelithiasis. She had a laparoscopic cholecystectomy. Post operative period was uneventful. Histology of the gallbladder showed fibrosis in the mucosa and schistosomal ova in the wall. As a conclusion we can see that due to the lack of specific clinical and radiological signs, the diagnosis of gallbladder schistosomiasis is established only after the histological examination.

## Introduction

Schistosomiasis is one of the most prevalent parasitic infections in the world, and it continues to be a global public health concern in the developing world. It affects almost 240 million people worldwide and causes more than 200.000 deaths per year [1]. In spite the fact of the high frequency of hepatic involvement particularly by the Mansoni species, schistosomiasis of the gallbladder is remarkably uncommon. The literature has reported less than ten cases most of which were associated with gallbladder lithiasis. And what makes this case even more interesting is the fact that Tunisia is no longer considered an endemic country [2].

## Patient and observation

**Figure 1:**
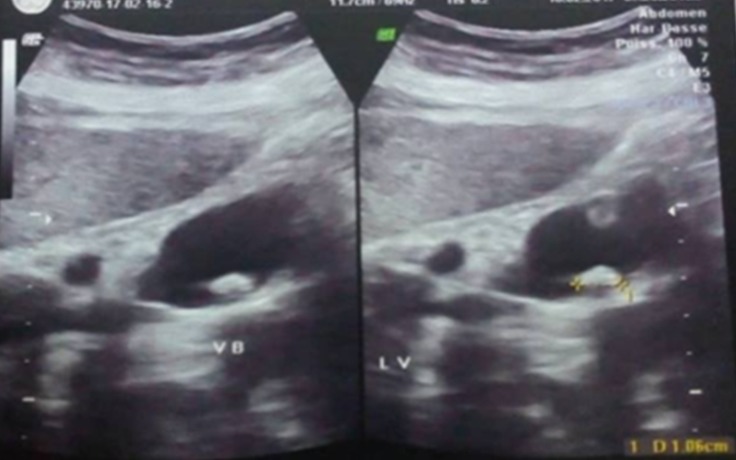
Undiluted gallbladder, thin-walled, with a stone of 1.06cm

**Figure 2:**
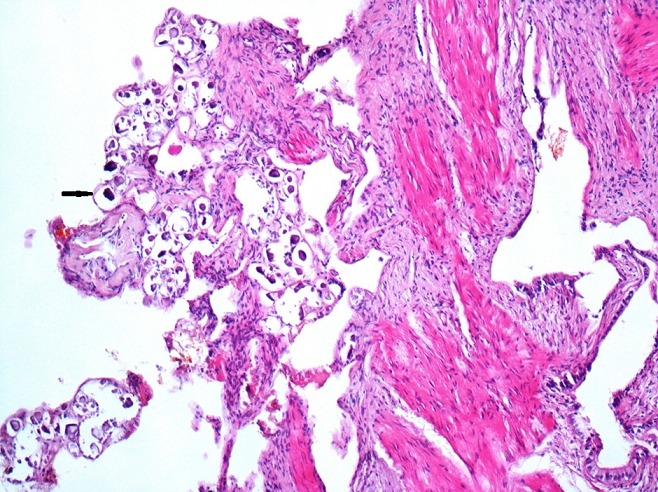
Calcified bilharzia eggs in the gallbladder chorion (HEx50)

**Figure 3:**
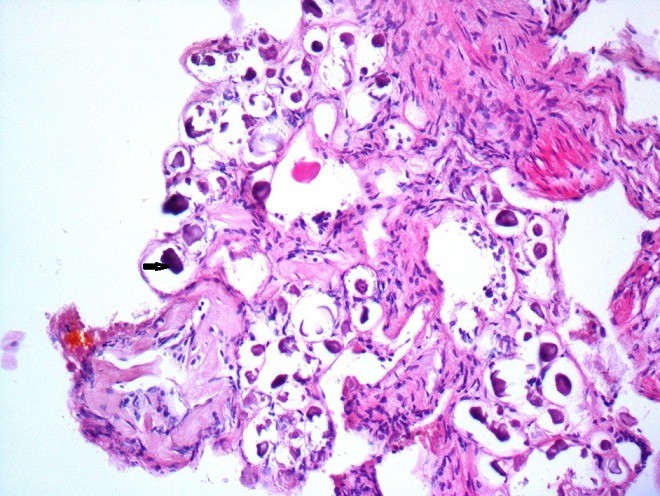
Eggs with hyaline thin wall sometimes calcified (HEx200)

**Figure 4:**
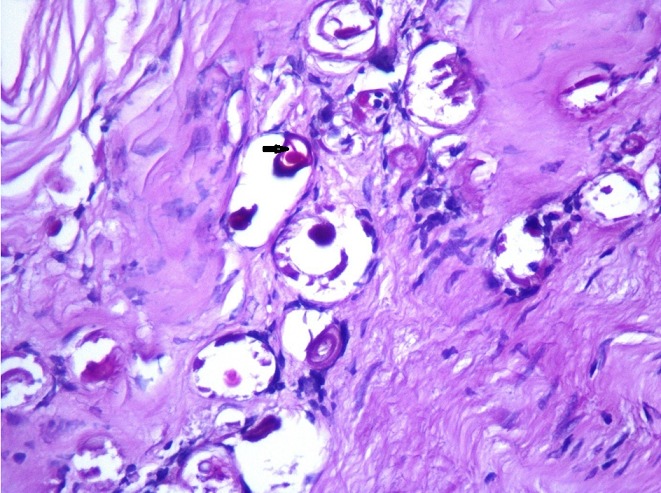
Colored eggs with periodic acid Schiff (PASx400)

We present the case of a 51 year-old woman from Béja, who lives in a village located in the north west of Tunisia. She has dyslipidemia and no surgical history. Note that the patient does not report any recent trips abroad especially in countries with high endemicity of Schistomasis. She presented with hepatic colic for 2 months duration. There were neither complaints about intestinal disorder, norchronic diarrhea, nor hematochezia, nor hematuria and nor burning urination. The physical examination showed a general good health, no jaundice, no fever, a flexible and painless abdomen and no visceromegaly. Laboratory tests showed no inflammatory signs and normal liver function tests. The abdominal Ultrasound showed a thin walled gallbladder with a 10mm stone and no other abnormalities (Figure 1). The patient was operated on using a laparoscopic approach. We found a slightly thick-walled gallbladder and a fine cystic duct. The cholecystectomy was uneventful. The patient was discharged the next day. Histologically there was inflammation and fibrosis in the mucosa, lymphocytic infiltrate in the chorion and calcified schistosomal ova were seen in the wall of the gallbladder which stained positively with periodic acid-Schiff (Figure 2, Figure 3, Figure 4). The patient received further examinations with a CT Urography that did not show any urinary localization. The decision was to refrain from additional medical treatment. Currently, at 12 months postoperative, clinical controls do not show recurrence or extra digestive localizations.

## Discussion

Schistosomiasis represents a global scourge and continues to pose a public health problem, especially in developing countries. It affects almost 240 million people worldwide and causes more than 200.000 deaths per year [1]. Despite to its high frequency, hepatic and particularly biliary involvement is rare; it's often associated with gallbladder vesicle. What makes this case even more interesting is the fact that Tunisia is no longer considered an endemic country [2]. Schistosomiasis is the most economically and socially destructive parasitic infection after malaria. Its origin is represented by a flat worm of the Helmint family [3]. The contamination occurs following the contact of its larval form with the skin through the water contaminated with the excrement containing parasite eggs. Inside the human body, the larva develops in adult schistosomes that live in blood vessels where females will release eggs. Usually the eggs will be externalized from the body by the stools, except some eggs that are able to persist in the tissue of the human body, thus causing the clinical manifestations of schistosomiasis [1, 3, 4]. This parasite can invade every organ, but it predominates especially in the colon, urinary bladder and ureter [1]. The explanation of this variation in incidence among organs is probably due to variation of the richness of venous drainage, the more veins are available, the more likely the female worms are to lay their eggs [5, 6]. The clinic is explained by the granulomatous immune response of the body to eggs of schistosomes. The pathogenesis of chronic schistosomiasis is almost exclusively explained by this granulomatous reaction. Schistosomiasis is therefore a widespread disease in underdeveloped countries, where access to drinking water remains a problem, Tunisia having eradicated this disease since 1983. All the cases of schistosomiasis are recorded and we recall no more than 10 cases per year which had all urinary location [2]. What makes this report even more interesting is the fact that the gallbladder schistosomiasis is extremely rare. In fact, less than 10 cases were described in the literature. Rappaport reported the first case in 1975 [6]. Then a serie of six patients was reported from Iraq in 1983 [7]. And in 1996, another case from Saudi Arabia was published [8].

Some speculations were made concerning this frequent combination. Some say that the fibrosis and the calcification of the cystic ducts, similar to that observed in the ureters of patients with urinary schistosomiasis, cause a stenosis. It is likely that this stenosis contributes to the stagnation of bile in the gallbladder and therefore to the formation of stones [7]. Others explains it by the fact that granulomatous lesions in the wall of the gallbladder make it thick which causes contractions of the gallbladder after fatty meals favouring the development of gallstones [8]. The symptoms are mainly epigastralgia and pain in the right upper quadrant. The abdominal ultrasound is the most practiced radiological exam but it is not contributive. It usually shows a thick walled gallbladder and the presence of cholelithiasis, also a hepatosplenomegaly with intense periportal fibrosis and portal hypertension can be seen when hepatosplenic involvement is present [9]. For gallbladder location of disease, the treatement remains surgical, represented by laparoscopic cholecystectomy. Post operative complementary medical treatment is only indicated if there are other localizations of the parasite. At surgery, the gallbladder is usually described as grey, irregular in thickness, infiltrating into the liver at its bed, the cystic duct can be thickened and the common bile duct is usually normal. The surgical specimen pathology usually reveals a lymphocytic infiltrate of the lamina propria, the chorion and the muscle, schistosomal ova can be found in mucosa, sub mucosa, fibro vascular coat or even free in the gallbladder content, but still, schistosomal granulomas of the gallbladder causing cholecystitis is very rare [10]. In the more chronic cases, ova are associated with a fibrocalcific reaction. Regarding medical treatment, there are several molecules: the praziquantel (Biltricide*) [10], which is taken in a single dose 40mg/kg. The side effects are mild and consist only of digestive disorders. The oxamniquine (Vansil*) is less used because of its multiple adverse effects. It should be noted that the treatment does not protect against reinfection which can reappear after 8 à 12 months, in addition to the probable existence of resistant parasitic strains. Preventive primary treatment remains the most effective solution. It will be necessary to educate the population on the hygiene of life and especially against swimming in dirty water [10].

## Conclusion

Gallbladder schistosomiasis is an extremely rare disease that should be suspected preoperatively in patients from endemic areas who develop symptoms suggestive of gallbladder affection. It remains a pathology with an atypical symptomatology with a histological discovery. The development of a vaccine is the best solution for eradication in addition to educating populations at risk for the hygiene measures that must be respected.

## Competing interests

The authors declare no competing interests.
